# Supplementary figures and images for: A 24-h activity profile and adiposity among children and adolescents: Does the difference between school and weekend days matter?
Source: PLoS One. 2023 May 18;18(5):e0285952. doi: 10.1371/journal.pone.0285952 (PMC10194946; doi:10.1371/journal.pone.0285952)

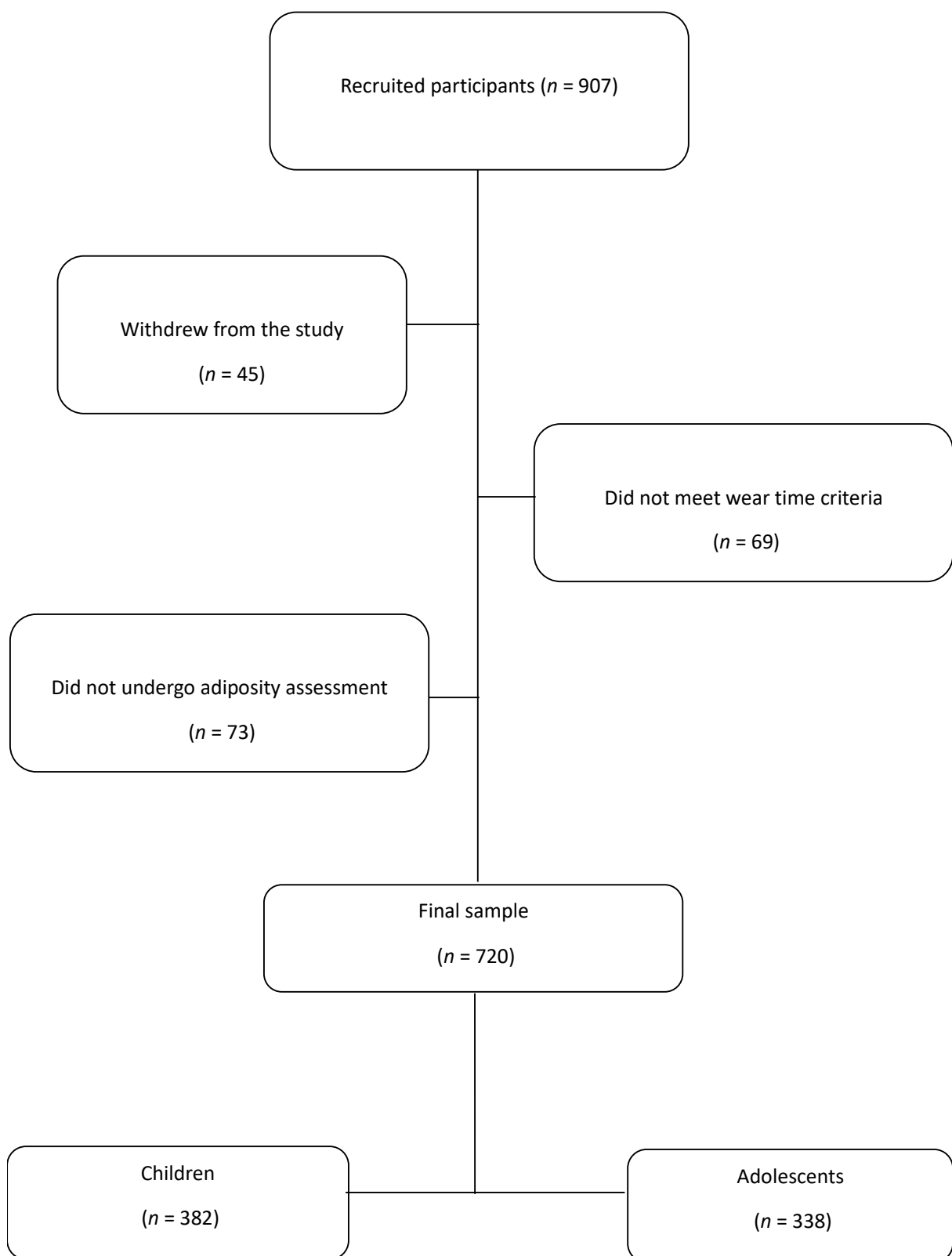

**S1 Figure 1.** Participant's exclusion flow chart

Supplement: S1 Fig — (PDF) [file pone.0285952.s002.pdf]
