# Supplementary material for: A 24-h activity profile and adiposity among children and adolescents: Does the difference between school and weekend days matter?
Source: PLoS One. 2023 May 18;18(5):e0285952. doi: 10.1371/journal.pone.0285952 (PMC10194946; doi:10.1371/journal.pone.0285952)
Supplement: S1 Appendix — (PDF) [file pone.0285952.s003.pdf]

S2 Table 1. Association between average acceleration and adiposity indicators in children, including the interaction between school and weekend days (n = 382)

	BMI *z*-score			Fat mass percentage (%)			Fat mass index (kg/m^2)[e]			Visceral adipose tissue (cm^2)		
	B	95% CI	*p-value*	*B*	95% CI	*p-value*	*B*	95% CI	*p-value*	*B*	95% CI	*p-value*
School days[a]												
AvAcc[b]	–0.002	–0.02, 0.01	0.653	**–0.09**	**–0.17, –0.001**	**0.047**	**–0.03**	**–0.05, –0.01**	**0.009**	**–0.53**	**–0.83, –0.23**	**<0.001**
Weekend days[c]												
AvAcc[b]	**0.01**	**0.002, 0.02**	**0.019**	0.08	–0.01, 0.14	0.067	**0.02**	**0.01, 0.04**	**0.014**	**0.29**	**0.04, 0.54**	**0.025**
Interaction between school and weekend days												
AvAcc	**–0.001**	**–0.002, –0.000**	**0.005**	**–0.005**	**–0.008, –0.001**	**0.029**	**–0.001**	**–0.002, –0.000**	**0.033**	**–0.017**	**–0.031, –0.002**	**0.021**

AvAcc – average acceleration, BMI – body mass index, CI – confidence interval, IG – intensity gradient.

[a] Model was adjusted for confounding factors and weekend days activity profile metrics.

[b] Model was additionally adjusted for interaction between school and weekend days.

[c] Model was adjusted for confounding factors and school days activity profile metrics.

[d]Generalized linear modelling with gamma family was used.

Boldface values denote significant association at $p < 0.05$.

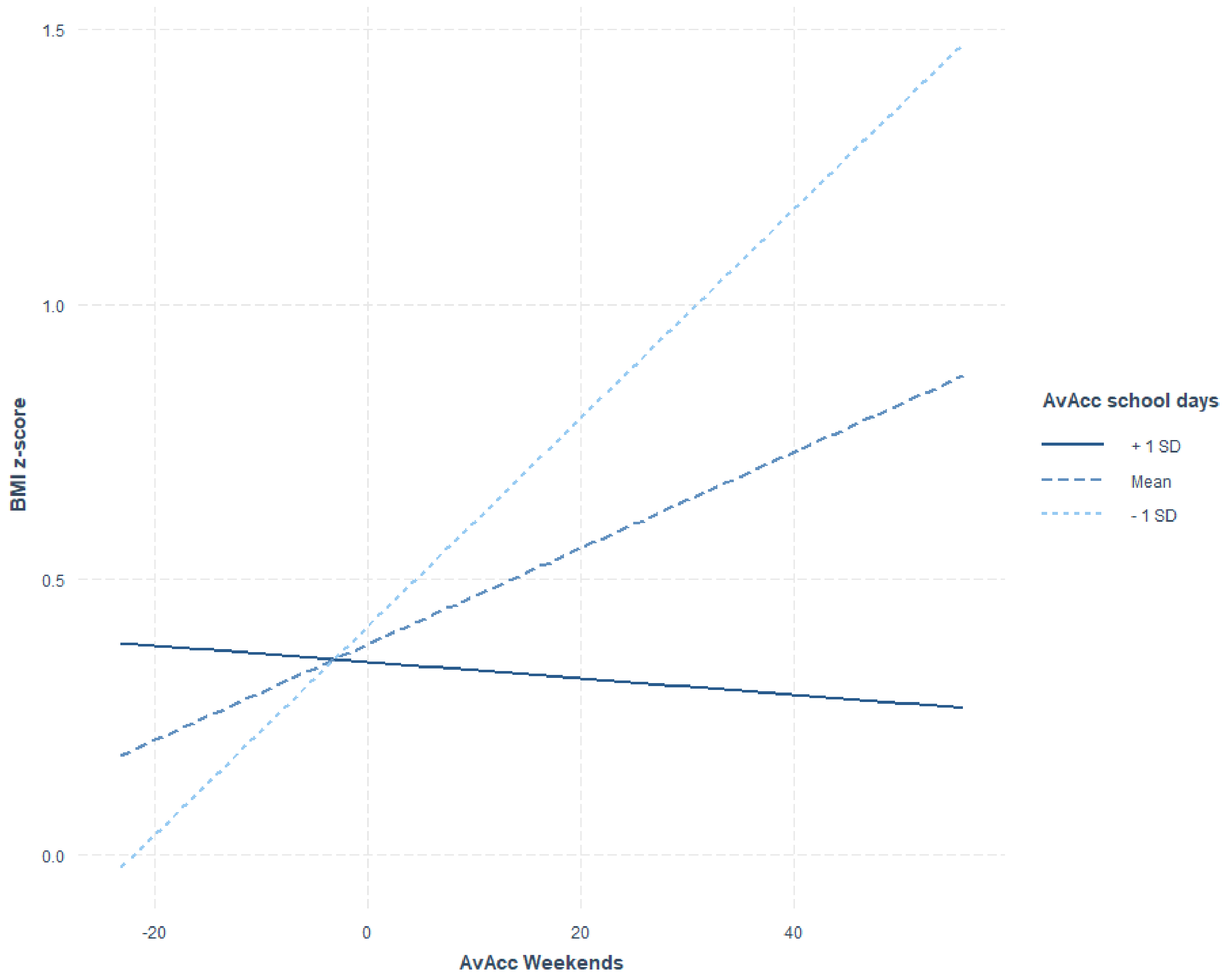

S2 Figure 1. Interaction between school and weekend days average acceleration and its effect on BMI *z*-score in children

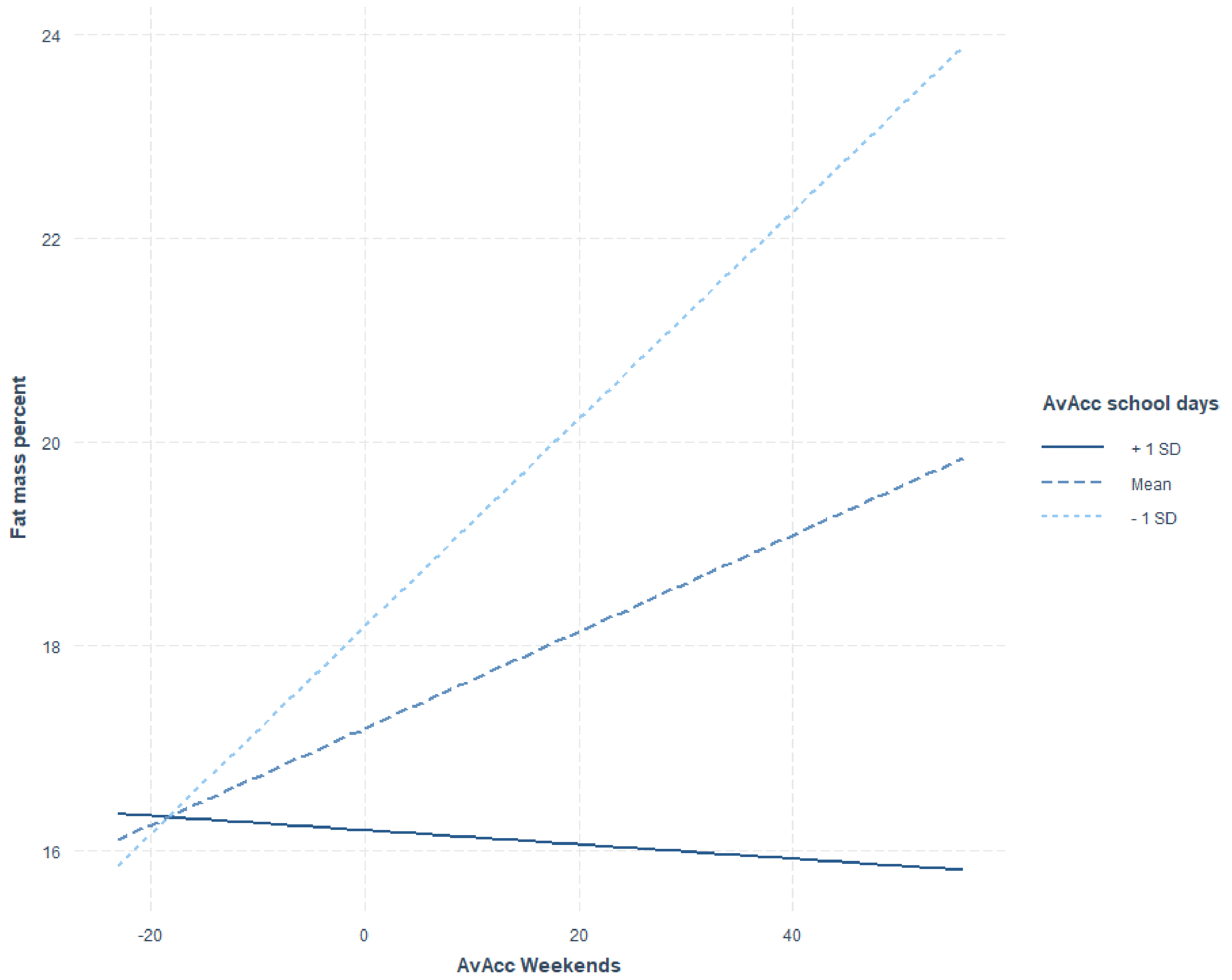

S2 Figure 2. Interaction between school and weekend days average acceleration and its effect on fat mass percentage in children

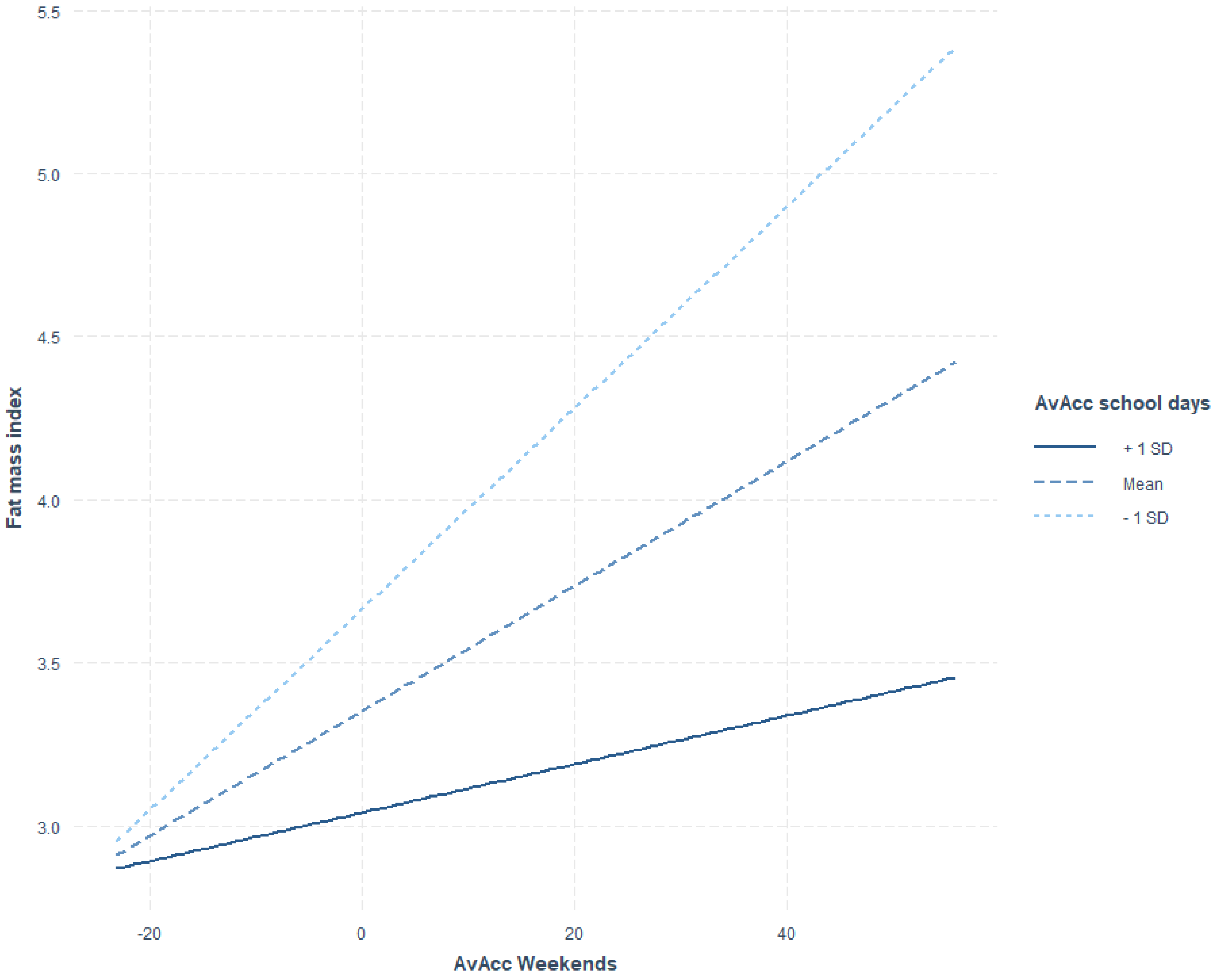

S2 Figure 3. Interaction between school and weekend days average acceleration and its effect on the fat mass index in children

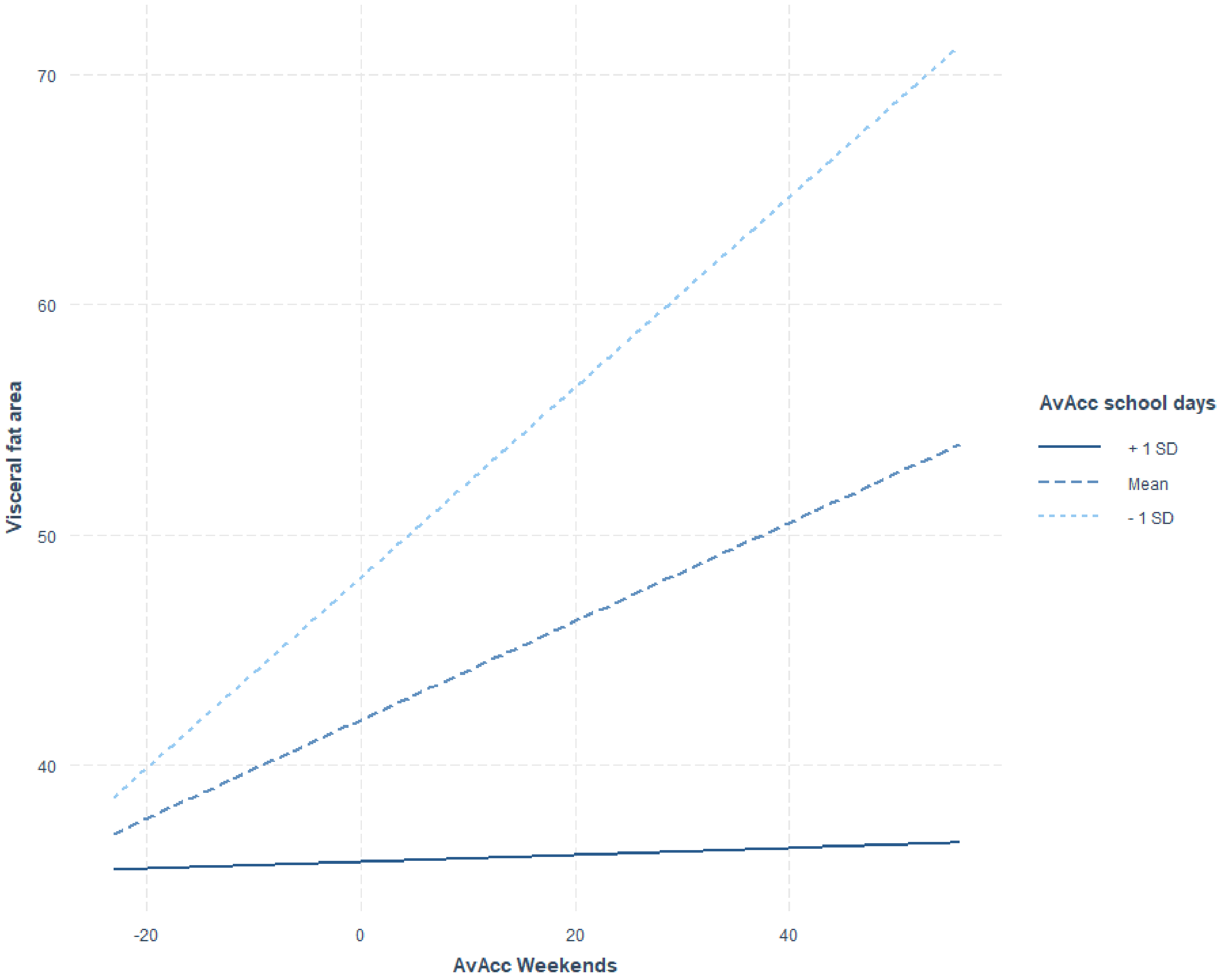

S2 Figure 4. Interaction between school and weekend days average acceleration and its effect on the visceral fat area in children

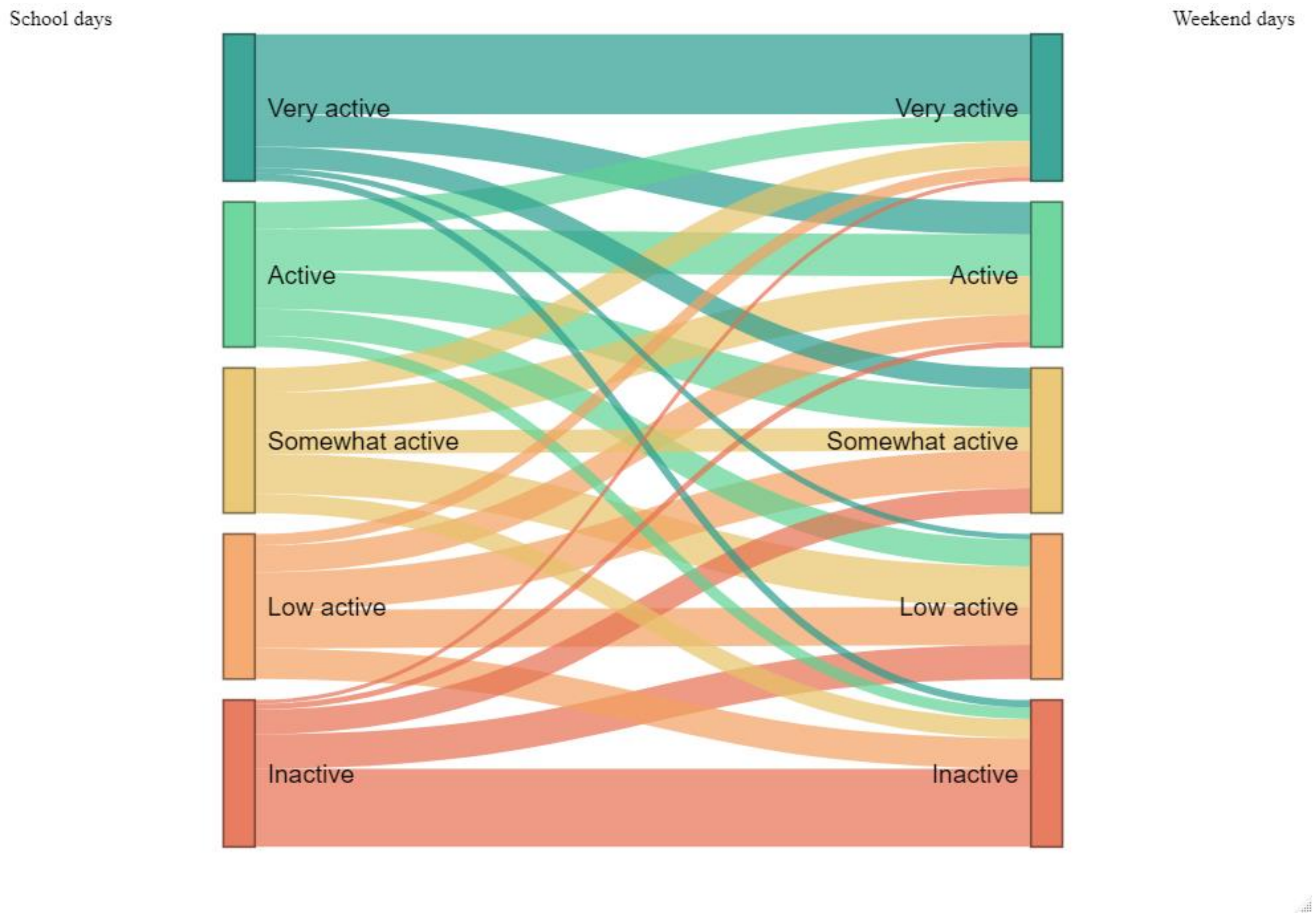

S2 Figure 5. The flow between pentiles of average acceleration between school and weekend days in children

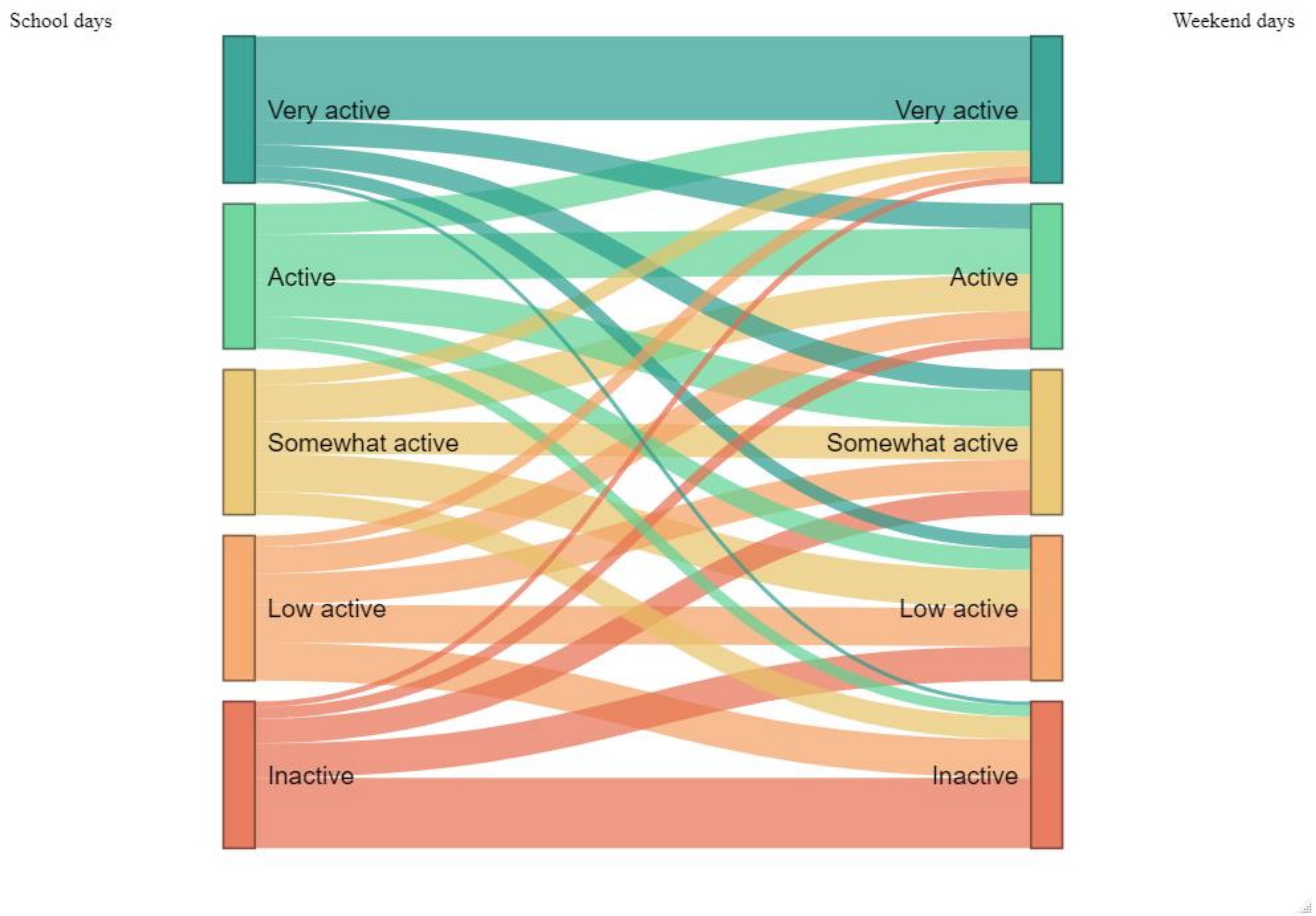

S2 Figure 6. The flow between pentiles of intensity gradient between school and weekend days in children
