# Supplementary material for: A 24-h activity profile and adiposity among children and adolescents: Does the difference between school and weekend days matter?
Source: PLoS One. 2023 May 18;18(5):e0285952. doi: 10.1371/journal.pone.0285952 (PMC10194946; doi:10.1371/journal.pone.0285952)
Supplement: Attachment — Submitted filename: Response_to_Reviewers.docx [file pone.0285952.s004.docx]

Dear Editors,

We are submitting a revised version of the manuscript “A 24-h activity profile and adiposity among children and adolescents: Does the difference between school and weekend days matter?” for publication in PLOS ONE. We would like to thank the editors and reviewers for their interest in our work and for the great comments that helped us to improve the manuscript. We addressed all the suggestions of the reviewers and provided point-by-point explanations below.

Sincerely,

**Aleš Gába**

*Corresponding Author*

# Reviewer 1

## Comment 1.1

In the methods section, kindly enumerate the inclusion and exclusion criteria for choosing your participants (creating a flow chart.

### Response

Thank you for the suggestion. We have added a line stating that the flow chart can be found in the supplementary file.

We have changed the text accordingly:

“ A total of 862 children (8–13 years) and adolescents (14–18 years) participated in the current study. Of all participants, 69 had to be excluded because they did not meet the accelerometer wear time criteria, and the other 73 did not undergo the adiposity assessment (S1 Appendix).”

## Comment 1.2a

This study used two accelerometers. What is the reason for this, and how many are used?

### Response

Thank you for your question. In our study, we used two research-grade accelerometers: Actigraph wGT3X-BT and GT9X Link. Actigraph wGT3X-BT was used for children, while the GT9X Link was employed for adolescents. This decision was made to achieve the highest compliance, especially for younger children, as the use of a nylon strap allows for proper attachment of the accelerometer even on very thin wrists.

## Comment 1.2b

Most studies are attached to the hip and thigh, but why did you put the accelerometers on the wrist? Also, please describe the measurement method in detail.

### Response

Thank you for bringing this to our attention. We have decided to use wrist-worn accelerometers because of superior compliance, compared to hip and thigh worn, respectively. Also, wrist-worn accelerometers are employed in three prominent large-scale studies, namely the UK Biobank, Whitehall II study, and NHANES, highlighting their widespread acceptance and utility in research settings. We have added the following sentence to the manuscript:

“An accelerometer worn at a non-dominant wrist was proven to be a valid measuring method with high compliance [23,24].”

Regarding the measurement method, in our text, we have added the following lines:

Lines 91.-93.: “Monitoring was carried out during weeks with a regular school schedule. Each participant received a monitoring device and was instructed on how to wear it. The devices were collected after eight days after their distribution. “

Line 117.: “The participants’ adiposity was assessed on the school premises in the morning, the day when the accelerometers were distributed.”

## Comment 1.3

Thank you for your suggestion. The typo has been corrected accordingly.

“(Biospace, Seoul, South Korea)”

# Reviewer 2

## Comment 2.1

“…were imputed by the multiple imputation method with 10 iterations to create five imputed datasets…” Why 10 iterations (and not e.g. 12)? Why five imputed datasets? Can you provided a reference for the imputation method since both dichotomous and continuous variables are imputed together?

### Response

Thank you for your question. We used 10 iterations, as it was proven to be sufficiently effective to provide precise data with missingness around 10% of the dataset. Our highest proportion of missing data per variable was under 7%. Also, our main variables of interest i.e. activity profile metrics and adiposity indicators included only observations with complete data. We have decided to impute only 2 confounding variables. Our decision was based on the following publications:

He, Y., Zhang, G., & Hsu, C.-H. (2021). Multiple Imputation of Missing Data in Practice: Basic Theory and Analysis Strategies (1st ed.). Chapman and Hall/CRC. <https://doi.org/10.1201/9780429156397>

Raghunathan, T. (2015). Missing Data Analysis in Practice (1st ed.). Chapman and Hall/CRC. <https://doi.org/10.1201/b19428>

## Comment 2.2

Maybe it would be still beneficial for the readers to explain the abbreviations LPA and MPA.

### Response

Thank you for bringing this to our attention. We have decided to omit the abbreviations. The sentence was reworded accordingly:

“Our recent longitudinal analysis also revealed that prolonged SB and vigorous PA increased, while time spent in light and moderate PA decreased during the transition from childhood to adolescence [36].”
